# Safety and Efficacy of Ritlecitinib for the Treatment of Patients with Alopecia Areata: A Systematic Review and Meta-Analysis of Controlled Trials

**DOI:** 10.3390/jcm14061817

**Published:** 2025-03-08

**Authors:** Samah Omar Ali Alfahl, Abdullateef Alzolibani

**Affiliations:** 1 Department of Family & Community Medicine, Taibah University, Janadah Bin Umayyah Road, Madinah 42353, Saudi Arabia; 2 Department of Dermatology, College of Medicine, Qassim University, P.O. Box 30109, Buraidah 51477, Saudi Arabia

**Keywords:** ritlecitinib, alopecia areata, systematic review, meta-analysis, controlled trials

## Abstract

**Background**: Alopecia areata (AA) typically presents as round patches of hair loss (e.g., scalp, eyebrow/eyelash, and body), has an unpredictable disease course, and may relapse and remit. AA is a condition with a lifetime risk of approximately 2% in the global population with an annual incidence rate ranging from 2.53 to 26 per 100,000. This comprehensive systematic review and meta-analysis was performed to determine the safety and efficacy of Ritlecitinib in patients with AA. **Methods**: A systematic search was conducted in PubMed and Cochrane CENTRAL Library for randomized controlled trials (RCTs). We used mean difference with 95% confidence intervals to assess the effectiveness and odds ratio to assess the safety profile. A total of 65 publications were identified through a database search. Following two stages of screening, we included 13 publications. All the studies were parallel and double-blind RCTs and published between 2020 and 2022. **Results**: Our analysis revealed a significant reduction in SALT score at week 12 and week 24 of (−17.43 [−24.67 to −10.20]; *p* < 0.0001) and (−20.95 [−29.01 to −12.89]; *p* < 0.0001), respectively, in patients treated with Ritlecitinib compared to placebo. Furthermore, a significant improvement in PGIC score at week 24 was observed. Additionally, Ritlecitinib revealed a slightly higher reduction in AASIS score compared to placebo; however, this difference was statistically non-significant. Notably, the Ritlecitinib group experienced a higher frequency of headaches, acne and nasopharyngitis compared to placebo, while the placebo group reported a greater occurrence of serious adverse events compared to the Ritlecitinib group. This higher rate of serious events in the placebo arm could be explained by the placebo effect, although these differences were statistically non-significant. **Conclusions**: These findings suggest that Ritlecitinib holds promise as an effective treatment for AA with an acceptable safety profile, warranting further investigation in larger cohorts and long-term studies.

## 1. Introduction

Alopecia areata (AA) is a chronic autoimmune disorder characterized by the acute onset of smooth, sharply demarcated, nonscarring, and patchy hair loss ranging from small circumscribed patchy areas on the scalp, which sometimes may progress to involve the entire scalp, face, and/or body [1,2,3,4]. AA is a condition with a lifetime risk of approximately 2% in the global population, with an annual incidence rate ranging from 2.53 to 26 per 100,000 [5]. Over the past two decades (1990–2019), the incidence of AA has increased by 49.14% [6]. Despite this significant rise, a slight decrease in age-standardized incidence rates has been observed. However, in some low-income regions and countries, the burden of AA has increased. Furthermore, females have a significantly higher age-standardized incidence rate than males [6]. The global prevalence of AA ranges from 0.02% to 0.21%, with a lifetime prevalence observed between 2.5% and 13.8%. Higher prevalence rates of AA are observed in high-income countries and in adults compared to children [5,7]. Alopecia universalis (AU) (total body hair loss), alopecia totalis (AT) (total scalp hair loss), alopecia in an ophiasis pattern (band-like hair loss on the temporal and occipital scalp), and ophiasis inversus (band-like hair loss in the frontoparietotemporal area) are the variants of AA [8]. Severity of AA is defined as follows based on SALT categories: no hair loss = 0%; mild/limited = 1–20%; moderate = 21–49%; severe = 50–94%; and very severe = 95–100% [9]. AA is commonly associated with psychiatric and medical comorbidities such as depression, anxiety, autoimmune thyroid disease, vitiligo, atopy, lupus erythematosus, psoriasis, and rheumatoid arthritis; specifically, severe and extensive forms of AA, such as alopecia totalis (AT) and alopecia universalis (AU), are often thought to have stronger associations with comorbidities [4,8,10]. Nail abnormalities are also associated with the disease, in which nail pitting is frequently observed [4,8,10]. Various internal stressors, encompassing both psychological and physiological factors such as adrenocorticotropic hormone (ACTH), corticosterone, and estradiol, are identified as etiological factors contributing to AA. Additionally, external environmental stressors, including infections, vaccinations, hormonal fluctuations, and dietary factors, may also play a role in the development of AA [8]. Treatment for AA encompasses a variety of options such as corticosteroids, immunotherapy, and other therapies, in which corticosteroids are widely regarded as the mainstay of treatment and can be administered topically, orally, or through injections. Topical or intralesional corticosteroids are usually regarded as the first-line therapy among them. Topical corticosteroids are preferred in adults and in children due to their ease of application and to avoid the severe discomfort associated with injections. Systemic corticosteroids are beneficial for refractory cases; however, they are used cautiously in children as they impede growth [1,8,11,12,13,14]. Immunotherapy, such as squaric acid dibutylester and diphencyclopropenone, serves as a second-line treatment for patients who do not respond to corticosteroids [1,8,11,13,14]. JAK-STAT inhibitors such as tofacitinib (JAK 1 and 3 inhibitors) and oral ruxolitinib are emerging potential treatments that have shown promising results when compared to conventional systemic treatments, but relapse rates have been observed. However, baseline investigations are recommended prior to the initiation of treatment [1,4,14]. Other treatments for AA include topical minoxidil, 5% solution or foam for adults and 2% for children, which promotes hair regrowth. Prostaglandin F2 alpha analogues are specifically used for eyebrow and eyelash AA. Steroid-sparing agents are frequently employed alongside systemic steroids or as monotherapy to prevent relapses of AA [1,11,14]. Supplementation with antihistamines such as fexofenadine and ebastine has demonstrated beneficial effects in AA associated with atopic dermatitis [13]. Additionally, topical contact irritants like anthralin followed by topical retinoids, phenol, salicylic acid, azelaic acid, tretinoin, and tincture iodine are used in children to induce localized irritation which in turn stimulates the immune response. Psoralen plus ultraviolet A radiation, or excimer laser therapy, and systemic immunomodulators are also utilized in the treatment of AA [1,11,14]. Early studies have revealed that a new emerging drug, Ritlecitinib, which belongs to the class of kinase inhibitors, is associated with a notably positive outcome in treatment of severe AA [15,16,17,18,19,20]. However, there remains a significant need for more extensive data to guide future research and clinical use. Given the lack of meta-analyses on Ritlecitinib, this study aims to conduct a systematic literature review (SLR) and meta-analysis to evaluate its efficacy and safety in patients with AA.

## 2. Materials and Methods

**Figure 1:**
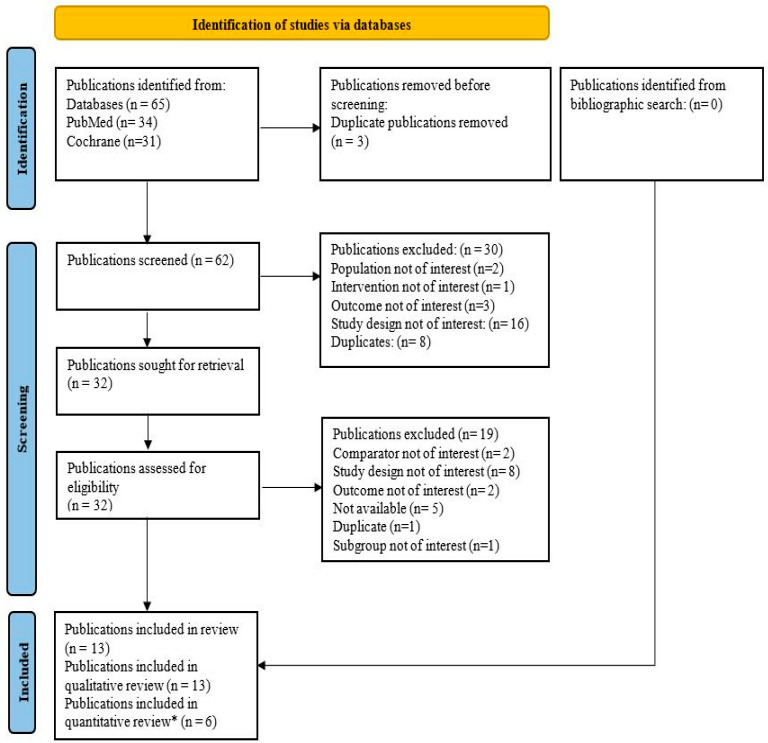
PRISMA flowchart of literature search and study selection. * In total, 6 publications were included in the meta-analysis, while the remaining 7 were the linked studies reporting from the same trial.

This meta-analysis was conducted in accordance with the Preferred Reporting Items for Systematic Reviews and Meta-Analyses (PRISMA) guidelines [21] (Figure 1).

### 2.1. Eligibility Criteria

The research question was structured using the PICOS (population, intervention, comparator, outcomes, study design) framework to define eligibility criteria. We included both published and unpublished randomized controlled trials (RCTs) that evaluated patients who had AA despite other therapies, irrespective of age, sex, country, and ethnic group. We also included studies assessing Ritlecitinib compared with placebo for the management of AA. We excluded non-human studies and in vitro research, phase 1 clinical trials, case reports, editorials, conference proceedings, commentaries, expert opinions, and reviews. We excluded studies lacking original data, non-randomized controlled trials, publications in languages other than English, duplicate reports, and trials without a control group for comparison with Ritlecitinib.

### 2.2. Search Strategy

A comprehensive literature search was conducted across multiple electronic databases, including PubMed and The Cochrane Central Register of Controlled Trials (CENTRAL), for relevant studies published from inception to January 2024. The search strategy employed the following terms: “alopecia areata” OR “alopecia universalis” OR “alopecia totalis” OR “alopecia circumscripta” OR “alopecia ophiasis” AND ‘‘ritlecitinib’’ OR “EAG4T1459K”. Detailed search strategies are provided in Supplementary Tables S1 and S2. To ensure comprehensive coverage, we conducted manual searches of reference lists from review articles, Google Scholar, and bibliographies to identify additional published trials. Clinical trial registries were also examined for unpublished studies. While no date restrictions were imposed, the electronic searches were limited to English-language publications.

### 2.3. Study Selection

Two authors (SA and AA) independently conducted first-pass screening (FPS) by reviewing the titles and abstracts of all retrieved records to identify potentially eligible articles based on predetermined criteria. Subsequently, full texts of articles deemed eligible during FPS were independently evaluated by the same two authors (SA and AA) in a second-pass screening (SPS) to determine final inclusion. Any discrepancies between the reviewers (SA and AA) during both FPS and SPS were resolved through discussion with a third reviewer.

### 2.4. Data Extraction and Management

Data extraction was independently performed by two authors (SA and AA) using standardized templates. All relevant information from the included randomized controlled trials (RCTs) was systematically extracted. Any discrepancies in data extraction were resolved through discussion with the third reviewer. The following details were extracted: study identification, authors details, study objectives, study design, setting of intervention, study population, measures, and main findings (SALT scores [a quantitative measure of scalp hair loss in alopecia areata. The scalp is divided into four areas, each assigned a percentage based on hair loss. Scores range from 0 (no loss) to 100 (complete loss).]; PGIC scores [a patient-reported outcome measure using a 7-point scale to assess perceived change in overall condition, ranging from “very much improved” to “very much worse”]; AASIS scores [a patient-reported measure for alopecia areata, evaluating the impact of symptoms on physical, emotional, and social aspects of a patient’s life]; eyelash assessment; eyebrow assessment; safety; etc.). The duration point was chosen because it was the most reported follow-up duration in the existing literature.

### 2.5. Methodological Quality Assessment of Included Studies

Two reviewers (SA, AA) independently assessed the methodological quality of the included studies using the Cochrane Collaboration’s Risk of Bias (RoB) assessment tool [22]. This tool evaluates six domains: selection bias, performance bias, detection bias, attrition bias, reporting bias, and other potential sources of bias. Each domain was categorized as low, unclear, or high risk of bias. Any discrepancies in the quality assessment were resolved through discussion with a third reviewer.

### 2.6. Statistical Analysis

All statistical analyses were conducted using R software version 4.2.2. For dichotomous outcomes, efficacy estimates were expressed as odds ratios (ORs). Continuous outcomes were reported as mean changes from baseline with corresponding 95% confidence intervals (CIs). Where not directly reported, standard deviations (SDs) were derived from standard errors or 95% CIs. In cases where SDs were not available, they were imputed following the methods outlined in the Cochrane Handbook for Systematic Reviews of Interventions [23]. The Higgins I^2^ statistic and Cochran’s Q test were used to assess the potential statistical heterogeneity among trials. The meta-analysis was conducted using a random-effect model (restricted maximum likelihood method) irrespective of low heterogeneity (<50%) or high heterogeneity (>50%). A funnel plot was not generated due to the low number of studies (<10) included [23].

## 3. Results

### 3.1. Current Study Characteristics

Through a comprehensive database search, a total of 65 publications were identified; among these, 3 duplicate publications were identified and subsequently removed. The remaining 62 publications underwent primary screening. Following a review of titles and abstracts, 30 publications were excluded. The remaining 32 publications were deemed potentially relevant and selected for full-text evaluation. Further, 19 publications were excluded as they were found to be irrelevant, insufficient, or ambiguous (Supplementary Table S3). Finally, 13 [15,16,17,18,19,20,24,25,26,27,28,29,30] publications were deemed relevant and included. Among them, six [15,16,18,20,24,25] publications having unique quantitative data were included in the meta-analysis.

All the included trials were published between 2020 and 2023 and included patients with ≥50% scalp hair loss. All the studies were parallel and double-blind RCTs. Furthermore, it is important to note that the patient population across these studies consisted of individuals with at least 50% scalp hair loss. The number of patients enrolled in each study varied, ranging from 46 [15] to 718 [19,25] participants. The average age of the patients was found to be 31.03 years and the majority of the patients were white, ranging between 47.4% [16] and 96% [20] across the studies. The duration of follow-up in these studies spanned from 24 to 48 weeks. Baseline characteristics of the publications are shown in Table 1.

### 3.2. Quality Assessment of Included Studies

**Figure 2:**
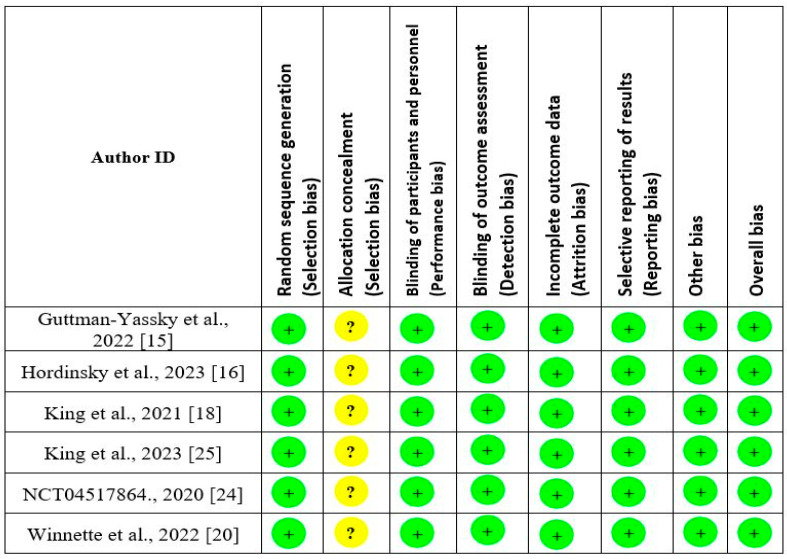
Assessment of the risk of bias in the included studies with the Cochrane domain-based quality assessment tool [15,16,18,20,24,25]. +, low risk; ?, unclear risk.

The risk of bias assessment encompassed all the publications included in the meta-analysis, as depicted in Figure 2. Each publication explicitly described the randomization process, but provided insufficient details regarding allocation concealment, which introduces a potential risk of selection bias. All studies were double-blinded, thereby eliminating the risk of performance and detection biases. They adhered to the intention-to-treat principle for outcome analysis, mitigating potential attrition bias. There was no indication of the selective reporting of results, ruling out reporting bias. The publications included their clinical trial registration information, disclosed conflicts of interest, and reported the source of funding to minimize other potential biases. Overall, all studies were found to have a low risk of bias.

### 3.3. Meta-Analysis

#### 3.3.1. Efficacy of Ritlecitinib on SALT Score

**Figure 3:**
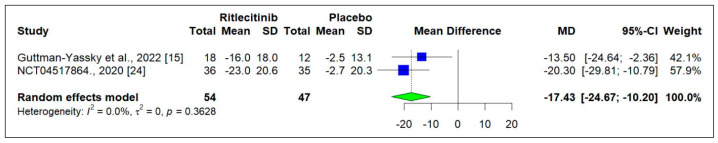
Change in SALT score from baseline to week 12 [15,24]. Square box indicated individual study estimates; Diamond box indicates overall pooled estimate.

**Figure 4:**
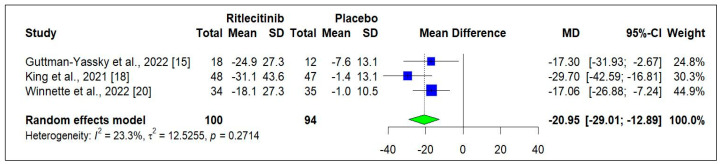
Change in SALT score from baseline to week 24 [15,18,20]. Square box indicated individual study estimates; Diamond box indicates overall pooled estimate.

Two randomized controlled trials (RCTs) [15,24] were analyzed to assess Ritlecitinib impact on the change in SALT score from baseline. Figure 3 illustrates the effectiveness using a random effects model, showing a significant reduction in SALT score at week 12 (−17.43 [−24.67 to −10.20]; *p* < 0.0001). Additionally, at 24 weeks, three RCTs [15,18,24] reported changes in SALT scores. Figure 4 visually demonstrates that Ritlecitinib exhibits superior efficacy with statistically significant results compared to placebo (−20.95 [−29.01 to −12.89]; *p* < 0.0001).

#### 3.3.2. Efficacy of Ritlecitinib on AASIS Score

**Figure 5:**
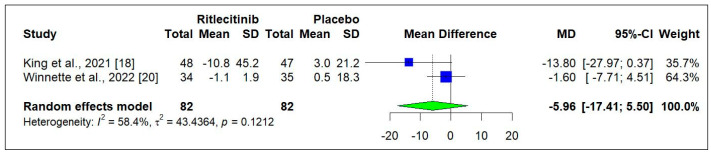
Change in AASIS score from baseline to week 24 [18,20] Square box indicated individual study estimates; Diamond box indicates overall pooled estimate.

Two RCTs [18,20] were analyzed to compare the impact of Ritlecitinib on the change in the Alopecia Areata Symptom Impact Scale (AASIS) scores. In Figure 5, the random effects model illustrates the effectiveness, revealing a slightly higher reduction compared to placebo, though this was statistically non-significant (−5.96 [−17.41 to 5.50]; *p* = 0.3082).

#### 3.3.3. Efficacy of Ritlecitinib on PGIC Score

**Figure 6:**
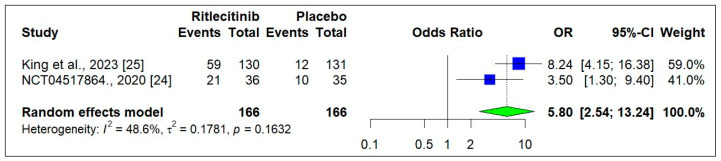
Change in PGIC score from baseline to week 24 [24,25]. Square box indicated individual study estimates; Diamond box indicates overall pooled estimate.

Two RCTs [18,24] were analyzed to evaluate Ritlecitinib impact on the proportion of patients who experienced an improvement in the Patient Global Impression of Change (PGIC) scores. Figure 6 demonstrates that the patients treated with Ritlecitinib reported significantly higher rates of moderate or great improvement in alopecia symptoms compared to placebo (5.80 [2.54 to 13.24]; *p* < 0.0001).

#### 3.3.4. Safety of Ritlecitinib

**Figure 7:**
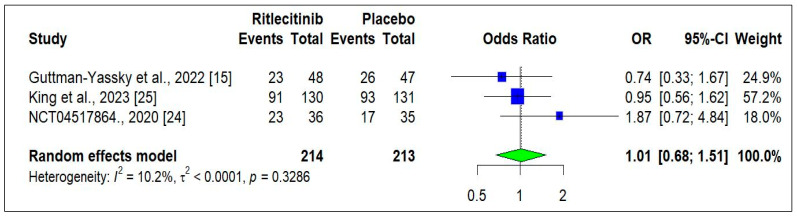
Total adverse events at the end of the study [15,24,25]. Square box indicated individual study estimates; Diamond box indicates overall pooled estimate.

Three RCTs [15,18,24] reported the safety-related data in patients treated with Ritlecitinib. The occurrence of adverse events was similar, and no statistical difference was observed between the Ritlecitinib and placebo groups (OR: 1.01, [0.68−1.51]; *p* = 0.9550) (Figure 7).

**Figure 8:**
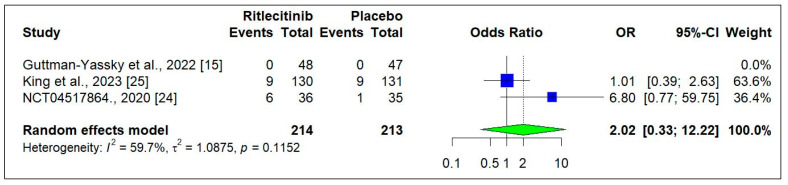
Treatment discontinuation due to adverse events at the end of the study [15,24,25]. Square box indicated individual study estimates; Diamond box indicates overall pooled estimate.

**Figure 9:**
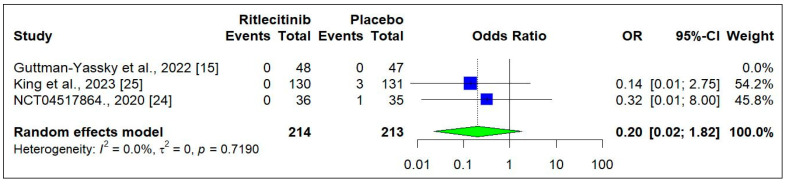
Total serious adverse events at the end of the study [15,24,25]. Square box indicated individual study estimates; Diamond box indicates overall pooled estimate.

Figure 8 shows that treatment discontinuation due to adverse events was relatively higher with Ritlecitinib compared to placebo, although this difference was not statistically significant (OR: 2.02, [0.33 to 12.22]; *p* = 0.440) [15,18,24]. Similarly, Figure 9 indicates that serious adverse events were slightly more frequent in the placebo group, but this difference was also not statistically significant (OR: 0.20, [0.02 to 1.82]; *p* = 0.1540) [15,18,24]. The incidence of headache, acne formation, and nasopharyngitis was higher with Ritlecitinib compared to placebo, though these differences were not statistically significant (headache, *p* = 0.7012; acne, *p* = 0.2323; and nasopharyngitis, *p* = 0.1197) (Supplementary Figures S1–S3) [15,18,24].

## 4. Discussion

Alopecia areata (AA) typically presents as round patches of hair loss (e.g., scalp, eyebrow/eyelash, and body), has an unpredictable disease course, and may relapse and remit. Several therapies are used off-label to treat AA, including corticosteroids and immunosuppressants [31]. Treatment options such as topical sensitizers and oral or topical minoxidil differ in tolerability, efficacy and/or safety. The oral Janus kinase (JAK) 1 and 2 inhibitor baricitinib is approved the United States (US), the European Union, and Japan to treat severe AA, but only in adults [16]. Ritlecitinib is a novel therapeutic agent that exhibits dual selectivity for the JAK3/TEC family kinase receptor, and it is approved in the US and Japan for the treatment of severe AA in patients 12 years and older [16]. Ritlecitinib has shown promising results for treating patients with AA. For instance, administering Ritlecitinib at doses of 50 mg and 30 mg daily for 24 weeks has led to notable hair regrowth, with a significant number of patients achieving a SALT score of 20 or lower (indicating ≤20% scalp hair loss) after 6 months, compared to those on a placebo [32]. Moreover, another study highlighted the benefits of Ritlecitinib for managing AA in patients with more than 50% scalp hair loss. This conclusion was based on observed changes in molecular biomarkers between weeks 12 and 24, suggesting a need for further evaluation of potential additional changes beyond 24 weeks [15]. Additionally, Ritlecitinib treatment was associated with a significant reduction in proinflammatory biomarkers and an increase in melanocyte products in the skin and blood of participants with non-segmental vitiligo (NSV), indicating its therapeutic potential. These changes correlated positively with the clinical response in AA [33]. Recent findings also indicate that Ritlecitinib is well tolerated and has an acceptable safety profile for up to 24 months in patients aged 12 years and older with AA [34]. Importantly, recent data also show that Ritlecitinib treatment does not result in an increased incidence of neurological or audiological adverse events [35]. In individual RCTs, Ritlecitinib has shown at least two grades of improvement from baseline in the eyelashes and eyebrows [16]. Similarly, patients treated with a loading dose of 200 mg for 4 weeks followed by 50 mg for 20 weeks reported greater improvement (67%) from baseline compared to 50 mg, 30 mg, or 10 mg for 24 weeks. Although Ritlecitinib has been the subject of numerous studies, a comprehensive systematic review synthesizing the available evidence has not yet been conducted [16]. In this study, we present a comparative assessment of Ritlecitinib’s efficacy and safety profiles relative to placebo, offering an in-depth analysis of the current clinical data. Our results shows that Ritlecitinib significantly reduced the SALT scores at week 12 (−24.67 to −10.20) and week 24 (−29.01 to −12.89). Also, PGIC scores showed that the patients treated with Ritlecitinib reported moderate or great improvement in their scores. These results are in line with recently published RCTs reporting similar reductions in SALT scores and improvements in PGIC scores at week 24 [15,16,18,19,20].

Our meta-analysis results demonstrated that the occurrence of adverse events was similar in both groups; however, treatment discontinuation was slightly higher for Ritlecitinib. Interestingly, serious adverse events were higher in the placebo arm, which could be explained by the placebo effect. Patients in the Ritlecitinib group experienced a higher incidence of headache, acne formation, nasopharyngitis, and upper respiratory tract infections compared to placebo; however, the differences were statistically non-significant [9,15,16,20,25]. Ongoing RCTs will confirm whether Ritlecitinib exhibits better long-term safety compared to placebo [36].

Current studies on Ritlecitinib show promising results for treating AA; however, several critical areas require further research. Future investigations should focus on conducting long-term trials to evaluate the sustained effectiveness and potentially delayed adverse effects of Ritlecitinib, given that existing studies have limited follow-up durations. Moreover, comparative analyses between Ritlecitinib and other JAK inhibitors, including baricitinib, would provide crucial insights into their respective efficacy and safety profiles. Identifying biomarkers linked to treatment response could significantly improve personalized therapeutic strategies. If long-term safety and efficacy are confirmed, Ritlecitinib has the potential to emerge as a first-line treatment for AA, particularly for patients who have not responded to conventional therapies. This advancement would require revisions to clinical guidelines and thorough cost-effectiveness studies to assess its economic impact relative to current treatments. These investigations should consider the potential for reducing disease burden and enhancing patients’ quality of life, thus influencing the future direction of AA management.

To the best of our knowledge, this study represents the first comprehensive systematic review and meta-analysis that integrates data from all Ritlecitinib phase 2 and phase 3 randomized controlled trials (RCTs) utilizing a placebo-comparative meta-analytical approach. Our aim was to provide a thorough evaluation of clinically relevant efficacy and safety outcomes. However, it is imperative to acknowledge certain limitations inherent in this study. Firstly, only a small number of eligible RCTs were identified. We conducted the meta-analysis using only published data available in journal articles or available on ClinicalTrials.gov. Secondly, due to limitations in the published data, we were unable to report pooled data on the effects of Ritlecitinib on eyelash assessment, eyebrow assessment, Alopecia Areata Patient Priority Outcomes (AAPPO) scores, and alopecia-related quality of life, and could not conduct subgroup analyses according to age, gender, and other factors for these outcomes. Finally, the duration of follow-up of the studies included in this meta-analysis was limited; therefore, further studies are needed to assess the efficacy and safety of Ritlecitinib in the long term.

## 5. Conclusions

Ritlecitinib, a novel therapeutic agent for AA, demonstrated significant efficacy in improving the condition by reducing the SALT and AASIS scores at weeks 12 and 24 compared to placebo, indicating a substantial improvement in hair regrowth. Additionally, patients reported moderate to great improvements in their PGIC scores, further supporting its clinical efficacy. Ritlecitinib presents a favorable efficacy profile while maintaining an acceptable safety profile, making it a promising therapeutic option for AA. These significant improvements in symptoms underscore the revival of hope in AA patients.

Future research for Ritlecitinib in AA treatment should focus on long-term safety and efficacy studies, and comparative studies with other JAK inhibitors. Investigating its effects in diverse populations, including pediatric patients and different ethnicities, is crucial. Additionally, identifying biomarkers to predict treatment response could lead to more personalized and effective strategies for AA patients.

## Figures and Tables

**Table 1:** Baseline characteristics of included publications.

Author ID	Year	Type of Publication	NCT ID	Study Design	Dose of Ritlecitinib ^#^	Blinded/Open	Study Duration	Total Number of Participants
Guttman-Yassky et al., 2022 [15]	2022	Journal article	NCT02974868	RCT	NR	Blinded	24 weeks	46
Hordinsky et al., 2023 [16]	2023	Journal article	NCT03732807	RCT	10 or 30 or 50 mg	Blinded	24 weeks	105
King et al., 2021 [18]	2021	Journal article	NCT03732807	RCT	50 mg	Blinded	24 weeks	95
King et al., 2023 [25]	2023	Journal article	NCT03732807	RCT	50 mg	Blinded	24 weeks	718
Hordinsky et al., 2022 * [17]	2022	Abstract	NCT03732807	RCT	10 or 30 or 50 mg	Blinded	24 weeks	105
King et al., 2022 * [19]	2022	Abstract	NCT03732807	RCT	10 or 30 or 50 mg	Blinded	48 weeks	715
Mesinkovska et al., 2022 * [26]	2022	Abstract	NCT03732807	RCT	10 or 30 or 50 mg	Blinded	24 weeks	718
Piliang et al., 2023 * [27]	2023	Abstract	NCT03732807	RCT	10 or 30 or 50 mg	Blinded	24 weeks	111
Senna et al., 2023 * [28]	2023	Abstract	NCT03732807	RCT	10 or 30 or 50 mg	Blinded	48 weeks	359
Sinclair et al., 2022 * [29]	2022	Abstract	NCT03732807	RCT	10 or 30 or 50 mg	Blinded	48 weeks	718
Soung et al., 2023 * [30]	2023	Abstract	NCT03732807	RCT	50 mg	Blinded	48 weeks	105
NCT04517864., 2020 [24]	2020	Clinical trial document	NCT04517864	RCT	50 mg	Blinded	24 weeks	71
Winnette et al., 2022 [20]	2022	Journal article	NCT04517864	RCT	50 mg	Blinded	24 weeks	95

* Publications not included in the meta-analysis; # a loading dose of Ritlecitinib 200 mg was given in all of the studies.

## Data Availability

Data are contained within the article and Supplementary Materials.

## Supplementary Materials

The following supporting information can be downloaded at: https://www.mdpi.com/article/10.3390/jcm14061817/s1, Supplementary Figure S1: Total headache events at week 24; Supplementary Figure S2: Total acne events at week 24; and Supplementary Figure S3: Total nasopharyngitis events at week 24. Supplementary Table S1: Search strategy for PubMed; Supplementary Table S2: Search strategy for Cochrane; Supplementary Table S3: List of excluded studies with reasons at step-2 level; Supplementary Table S4: PRISMA checklist.

## Abbreviations

The following abbreviations are used in this manuscript:

AA	Alopecia areata
AU	Alopecia universalis
AT	Alopecia totalis
RCTs	Randomized controlled trials
PRISMA	Preferred Reporting Items for Systematic Review
PICOS	Population, intervention, comparison, outcome, and study design
FPS	First-pass screening
RoB	Risk of bias
CI	Confidence interval
SD	Standard deviations
AASIS	Alopecia Areata Symptom Impact Scale
PGIC	Patient Global Impression of Change
US	United States
JAK	Janus kinase
NSV	Non-segmental vitiligo
AAPPO	Alopecia Areata Patient Priority Outcomes
